# The Emerging Role of Macrophages in Immune System Dysfunction under Real and Simulated Microgravity Conditions

**DOI:** 10.3390/ijms22052333

**Published:** 2021-02-26

**Authors:** Yulong Sun, Yuanyuan Kuang, Zhuo Zuo

**Affiliations:** Key Laboratory for Space Biosciences & Biotechnology, Institute of Special Environmental Biophysics, School of Life Sciences, Northwestern Polytechnical University, Xi’an 710072, China; kuangyuanyuan@mail.nwpu.edu.cn (Y.K.); zuozhuo@mail.nwpu.edu.cn (Z.Z.)

**Keywords:** macrophages, microgravity, TNF-α, arginase I, ICAM-1

## Abstract

In the process of exploring space, the astronaut’s body undergoes a series of physiological changes. At the level of cellular behavior, microgravity causes significant alterations, including bone loss, muscle atrophy, and cardiovascular deconditioning. At the level of gene expression, microgravity changes the expression of cytokines in many physiological processes, such as cell immunity, proliferation, and differentiation. At the level of signaling pathways, the mitogen-activated protein kinase (MAPK) signaling pathway participates in microgravity-induced immune malfunction. However, the mechanisms of these changes have not been fully elucidated. Recent studies suggest that the malfunction of macrophages is an important breakthrough for immune disorders in microgravity. As the first line of immune defense, macrophages play an essential role in maintaining homeostasis. They activate specific immune responses and participate in large numbers of physiological activities by presenting antigen and secreting cytokines. The purpose of this review is to summarize recent advances on the dysfunction of macrophages arisen from microgravity and to discuss the mechanisms of these abnormal responses. Hopefully, our work will contribute not only to the future exploration on the immune system in space, but also to the development of preventive and therapeutic drugs against the physiological consequences of spaceflight.

## 1. Introduction

Spaceflight has greatly expanded human understanding of the space environment and has made significant progress in space research. However, various systems of the human body exposed to the space environment will adapt and adjust accordingly, such as reprograming the immune system [1,2]. Moreover, there is growing evidence indicates that the immune system is dysregulated in space, which subsequently leads to a series of severe physiological consequences [3]. Early experiments regarding the dysregulation of the immune system in space can be dated back to the 1970s, as more than half of the astronauts (15 of the 29) showed a depressed immune system during spaceflight or after return to Earth in the Apollo missions. As a result, some astronauts suffered from bacterial or viral infections [4]. In later research, profound changes in the cells of the immune system are reported (Table 1 and Table 2) [5,6,7], including (i) the production of cytokines, (ii) the distribution of leukocyte subsets, (iii) the activation of T cells, and (iv) the function of granulocytes and natural killer cells. Besides, microgravity-induced immune system alterations make the host more susceptible to the pathogen invasion [8,9,10].

Currently, study of the effects of spaceflight on human and animals reveal that microgravity is an important factor that affects the immune system [11,12]. These studies point out that the influence of microgravity on the immune cells mainly includes: (1) Changing the transduction of intracellular signaling pathways; (2) applying gravity directly to cells [13]. As one of the indispensable components of the body’s immunity, macrophages play a pivotal role in immune disorders under microgravity conditions [14,15]. Currently, there is growing evidence that regulating polarization is a way for microgravity to affect macrophages function [16,17,18].

Derived from monocytes in the blood, macrophages are characterized by phenotypic variability and functional diversity. It is believed that there are two phenotypes of macrophages: Classically activated macrophages and alternatively activated macrophages [19]. Classically activated macrophages (also called M1) are activated by IFN-γ/TLR ligands and play critical roles in the body’s immune responses, such as clearing pathogenic microorganisms and producing inflammatory factors [20]. Alternatively activated macrophages (also called M2) are induced by IL-4/IL-13 and are characterized by promoting tissue repair, alleviating inflammation, and highly expressing arginase I [21]. Under normal gravity conditions (1 g), the balance between M1 and M2 macrophages is of great significance in immune regulation. Under microgravity conditions, the expression of M1 and M2 macrophages markers are significantly changed [16,17]. However, the mechanism of microgravity-induced macrophage dysfunction has not been fully clarified. Therefore, a systematic summary of macrophages regulation under microgravity conditions is an issue worthy of attention.

In the present study, we summarize the expression of macrophage polarization markers (M1, TNF-α; M2, arginase I) under microgravity conditions and identify their related signal transduction. Hopefully, our work will contribute not only to the future study of immune dysfunction in microgravity, but also to the development of preventive and therapeutic approaches for spaceflight.

## 2. Comparison of Facilities Used for Microgravity Research

Currently, the best way to investigate the effects of microgravity on the immune system is to conduct experiments in space. However, the opportunities for spaceflight experiments are scarce and costly, which greatly limits the development in this field. Based on the above reasons, many microgravity simulators have been developed. At present, the microgravity simulators that have been widely used include parabolic flight, two-dimensional (2-D) clinostat, random positioning machine (RPM), and rotating wall vessel (RWV) [22,23]. Given that only a few data were available in this field, we discussed the principles and applications of various simulation approaches (including their advantages and disadvantages), which may involve different cell lines.

### 2.1. Parabolic Flight

Parabolic aircraft is a device that utilizes the changes in the aircraft’s flight trajectory to mimic a microgravity environment. In general, a complete parabolic flight consists of four phases. Firstly, the aircraft accelerates in horizontal flight. Subsequently, the aircraft is pulled up to approximately 45 degrees. Next the aircraft is pushed over the top and enters the weightlessness phase, which requires constant adjustments to zero lift. Finally, the lever is pulled up quickly and resumed horizontal flight [24]. The weightless time created by a parabolic flight is about 20–30 s, and the parabolic flights are often used to investigate rapid biological events, including hormone secretion and signal transduction [25].

In an experiment simulating Mars flight, participants were isolated and confined for 520 days. To investigate the body’s response to acute stress, all of them participated in a parabolic flight (A300 Zero-G; 90 min, 30 times × 20 s/time) after six months of rest. Participants’ saliva, urine, and ECG were systematically analyzed. The results showed that the cortisol level in the saliva of the experimental group was greatly increased during the parabolic flight (*p* < 0.0001) compared with the control group (not participating in simulated Mars flight). As for the EEG index, the beta activity of EEG in the experimental group was significantly enhanced (*p* < 0.01), while the alpha activity showed no marked difference. Besides, the level of adrenaline was dramatically increased in the urine of the participants (*p* < 0.001) [26]. The parabolic flight is also used in the study of macrophages under microgravity conditions. In a study exploring the function of macrophages during the parabolic flight (KC-135, 40 times × 20–30 s/time), researchers used the weak electric field to detect the spreading of B6MP102 bone marrow-derived macrophage. The results revealed that macrophages respond to microgravity within 8 s [27]. In summary, the parabolic flight has a good effect in the field of microgravity research, especially in responding to weightlessness-caused acute stress responses. However, the parabolic flight method also has certain limitations in the field of microgravity research, including high flight costs, short microgravity time, and flight conditions that are easily affected by the external environment [25].

### 2.2. 2-D Clinostat

The principle of 2-D clinostat to simulate microgravity environments, a device with one rotation axis, is to quickly and continuously rotate the samples to avoid the perception of gravity. Based on the above experimental conditions, the relative movement of samples can be neglected over time, thereby achieving a microgravity environment.

The production of ROS in macrophage has been extensively investigated under microgravity conditions. These studies reveal that the production of ROS in alveolar macrophages is a gravity-dependent process [14]. In an experiment using 2d-clinostat as a microgravity analog, the rotation speed of the device significantly affected the results. At a rotation speed of 60 rpm, the production of ROS (indicated by luminescence luminol assay) in alveolar macrophages (NR8383) was inhibited compared to the control group. This is consistent with the findings of earlier studies [14]. However, at a rotation speed of 2 rpm, there was no obvious difference in ROS production between the 1 g and microgravity group [28]. In addition, in the research of plant growth under microgravity conditions, 2-D clinostat has been proven to be a satisfactory simulator. In an experiment using a plant cell (Chara Rhizoid) as an object, microgravity exposure caused the statoliths to move away from the tip, which is consistent with the phenomenon observed in real microgravity [22]. Based on the above evidence, the speed of the 2-D clinostat has a huge impact on the experimental results. Besides, 2-D clinostat can be used as a good microgravity simulation device when investigating slow physiological processes such as plant growth.

### 2.3. Random Positioning Machine

Based on the 2-D clinostat, the researchers further develop a device with two independently rotating axes. The term ‘‘3-D clinostat’’ is used to describe the condition where two axes are running with constant speed and direction. When two axes are running at different speeds and directions in operational mode, it is generally regarded as RPM. The principle of microgravity condition created by RPM is to rotate the sample at random speed and direction, which causes the gravity vector to be almost zero over time [22,23]. Therefore, the sample’s perception of gravity can be neglected.

Currently, RPM has been widely used in the microgravity field. For cells in the mononuclear-macrophage system, U937 cells exhibited reduced proliferation in RPM (40% slower than ground control; standard errors are <10%). This is consistent with the results obtained in spaceflight [29]. As for human endothelial cells (EA.hy926 cell line), the formation of tube-like aggregates was observed in both spaceflight and RPM (60°/s, real random mode), but not in the ground 1 g control. Moreover, in the process of 3D structure formation, the expression of IL-6 was up-regulated (*p* < 0.05) [30]. These results suggested that IL-6 may be involved in the formation of 3D-structure induced by microgravity. However, the device also has certain limitations in simulating microgravity. For example, the shear force and vibration of the device resulted in more variant forms of ROS production in macrophages, which is inconsistent with the results obtained in parabolic flight and 2-D clinostat [28].

RPM has been widely used in simulated microgravity experiments involving the cultivation of mammalian cells [31]. In the experimental system using RPM to simulate microgravity, the quality of microgravity obtained by the simulation is closely related to the specific parameters of RPM. The speed cannot be too high because the centrifugal force will become effective [32]. Moes and colleagues investigate the membrane, and actin dynamics of A431 cells used an RPM, and the parameters set were: Random direction, random speed, random interval, and the maximum random speed was 360°s^−1^ [33]. Considering that the main principle of RPM is to change the direction of gravity on the axis of rotation, RPM is suitable for biological systems with slow sensing processes. Otherwise, in a cell experiment system with a fast and sensitive sensing process, RPM may cause cells to be continuously stimulated by mechanical force and trigger stress responses and even cell death [22,32]. In addition, RPM is not quite suitable for animal behavior study as long as the speed is too low to disorient the animals [22].

### 2.4. Rotating Wall Vessel

The rotating wall vessels (also known as rotating cell culture system, RCCS) are initially developed by NASA for cell culture. The principle of this device is to balance the gravity vector by rotating the vessel so that the sample keeps in a static state relative to the vessel. Notably, the RCCS-created microgravity environment is characterized by extremely low shear forces on the sample.

At present, RCCS has been widely used in the research of microgravity exposure-caused immune dysfunction. In 2015, Wang et al. used RCCS (15 rpm) to investigate the effect of simulated microgravity on neutrophils function (ELISA assay). In this experiment, they observed increased secretion of nitric oxide (NO), interleukin-6 (IL-6) (*p* < 0.05), and interleukin-8 (IL-8) (*p* < 0.01) in DMSO-differentiated HL-60 cells, which is consistent with increased neutrophil cytokines secretion during spaceflight [34]. This finding suggested that cellular immune responses are enhanced under microgravity conditions. However, several studies on the immune activity of lymphocyte cultured in RWV show that lymphocyte activity and the responses to stimuli (PHA and ConA) are inhibited [35,36].

Overall, these ground-based facilities have been widely used in microgravity research and have made significant progress. It is worth noting that these ground-based microgravity simulators (such as clinostat, RCCS) also have some defects in simulating the microgravity environment: Some other factors (including shear and vibration) are introduced into the system, which may affect the results of the microgravity experiment [22,23]. Table 3 lists the facilities that applied to the microgravity research and associated parameters. In addition to conventional clinostat, special clinostats have also been developed to satisfy specific experimental demands, such as online kinetic measurements, microscopic observations, and even underwater detection [22]. Although the space environment is more complicated than ground-based microgravity facilities, these ground-based microgravity facilities have been proven to be satisfactory models for some specific samples. Furthermore, these models have been known to cause fluid shifts, muscle atrophy, bone loss, and immune dysfunction, which is similar to effects observed during spaceflight. In summary, researchers should be careful in choosing the models according to the different experimental purposes.

**Table 1:** Summary of changes in cytokines expression in space and ground-based microgravity facilities.

Item	Modeled Microgravity	Real Microgravity	Cell	Levels	Reference
IL-1	RWV		U937 (Human)	Increased	[37]
		During spaceflight	B6MP102 cells (Murine)	Increased	[38]
		Spacelab	PBMC (Human)	Decreased	[5]
		Postflight	PBMC (Monkey)	Decreased	[39]
IL-2		Postflight	Whole blood-T cell	Decreased	[40]
	RWV		U937 (Human)	Increased	[37]
IL-6		During spaceflight	PBMC (Human)	Decreased	[41]
		Postflight	Blood-monocytes (Human)	Decreased	[42]
	RCCS		Macrophages (Murine)	Increased	[16]
IFN-α		During spaceflight	Lymphocytes (Human)	Increased	[43]
		During spaceflight	Spleen cells (Murine)	Increased	[44]
IFN-β		During spaceflight	Lymph node T cells (Murine)	Increased	[43]
IFN-γ		During spaceflight	peripheral blood lymphocytes (Human)	Increased	[44]
		Postflight	Splenocytes (Rat)	Decreased	[45]
TNF-α		During spaceflight	Peripheral blood(Human)	Decreased	[41]
		During spaceflight	B6MP102 cells (Murine)	Increased	[38]
		Postflight	Whole blood (Human)	Decreased	[46]
	RCCS		Macrophages (Murine)	Decreased	[17]

PBMC, peripheral blood mononuclear cells.

**Table 2:** Summary of immune cells alterations in space or ground-based microgravity facilities.

Cell	Modeled Microgravity	Real Microgravity	Cell Location	Alterations	References
Lymphocyte	RWV		Lymph nodes (Mouse)	Abrogated antigen-specific function	[47]
		Spacelab	Blood (Human)	Inhibited response to mitogen Con A	[48]
	RWV		Peripheral blood (Human)	Inhibited locomotion, blunted ability to respond to PHA	[35]
	RWV		PBMC (Human)	Suppression of PHA activation	[36]
		Postflight	PBMC (Human)	Reduction of activity	[49]
Natural killer cell		Postflight	PBMC (Human)	Suppressed cytotoxic	[49]
		Postflight	Peripheral blood (Human)	Lower cell counts	[50]
		Spaceflight	Spleen (Rat)	Inhibited cytotoxicity	[51]
Neutrophil		Postflight	Blood (Human)	Increased number	[45,52]
		Postflight	Peripheral blood (Human)	Increased number	[52,53]
		Postflight	Circulating leukocyte subsets (Human)	Increased number	[54]
		Postflight	Blood (Human)	Increased number, lower phagocytosis, and oxidative burst capacities	[49,55]
Monocyte/ macrophage		SLS-1	Blood (Human)	Increased number	[56]
		Parabolic flight	BMDM (Mouse)	Enhanced proliferation, inhibited differentiation	[27]
		Postflight	Blood (Human)	Monocytopenia	[57]
		Postflight	Spleen (Rat)	Decreased number	[58]
		Postflight	Peripheral blood (Human)	Increased number	[53]
		Postflight	Peripheral blood leucocytes (Human)	Increased number	[59]
	RCCS		Spleen (Mouse)	Decreased number	[60]
		Postflight	PBMC (Human)	Reduction in phagocytosis	[49,61]

BMDM, bone marrow-derived macrophage; Con A, concanavalin A; PHA, phytohemagglutinin.

**Table 3:** Synopsis of commonly used facilities for microgravity research.

Devices	Principle	Application	Characteristic	Shortcoming	References
RPM	Randomizing the gravity vector direction and the gravity vector is averaged to nearly zero over time	Osteoblasts; T lymphocytes; adherent cells	Two axes with different speeds and directions	Cell behavior affected by the shear forces and other forces; no gas change	[29,62]
2-D Clinostat		Plants; small organism; unicellular; slow responsive living objects	One axis with fast and constant rotation	Vibration and centrifugal forces may lead to artifacts; no gas change	[63,64,65,66]
RWV (RCCS)		Suspended and anchorage-dependent cells; cell differentiation	Co-culture multiple cell types in a 3D spheroid morphology with low shear force	Lack of measurability; limited transfer of matter; additional environmental conditions such as the mixture	[67,68,69]
Parabolic Flight	Centrifugal forces counteract the gravity vector	Fast events, such as signal transduction, hormone secretion, binding of ligands to cell membranes	By controlling acceleration, creating a centrifugal force; about 25 s microgravity time	External conditions are not easy to control; high cost; short time of microgravity simulation	[25]

## 3. Macrophages under Microgravity Conditions

Macrophages are widely present in most tissues and have the functions of phagocytosis, antigen presentation, and secretion of various cytokines. Therefore, macrophages play important roles in regulating physiological processes such as immunity, repair, and metabolism. On the one hand, as the first line of the innate immune defense, macrophages patrol through the body and identify self and nonself-substances. When nonself-matter was identified, macrophages surround the pathogen and eliminate it by oxidative burst reaction or phagocytosis [70]. On the other hand, macrophages also participate in the process of activating specific immune response through antigen presentation, thereby maintaining the body’s homeostasis. Overall, the precise regulation of macrophage function is of considerable significance to the body’s physiological activities.

Under microgravity conditions, the dysfunction of macrophages has been widely reported and attracted a lot of attention. Microgravity-related researches suggest that macrophage dysfunction is a crucial determinant in weightlessness-induced immune disorder. Compared with the 1 g control, the macrophages’ numbers and the cytokines production were significantly changed in microgravity [11,71]. In addition, a series of abnormalities in cells of the monocyte-macrophage system in real microgravity were also observed, including reduction of cell number and oxidative burst reaction [14,61], decreased expression of human leukocyte antigen, alteration of the cytoskeleton, and differentiation-associated gene expression [72,73]. Notably, microgravity-induced changes in macrophages may break the balance between the pro-inflammatory and the anti-inflammatory system [74], which in turn affects a series of macrophages immune responses, such as migration, adhesion, and antigen presentation. Collectively, the dysfunction of macrophages is one of the underlying mechanisms of microgravity-induced immune dysregulation.

There are two types of factors that can affect the function of macrophages in microgravity: (1) Inflammatory mediators such as TNF-α and arginase I, where the former has pro-inflammatory effects and the latter has anti-inflammatory functions [20,21]; and (2) intercellular adhesion molecule 1 (ICAM-1), which is an essential medium for the interaction between white blood cells [75].

### 3.1. TNF-α

TNF-α, mainly secreted by activated macrophages, is an important mediator in the inflammatory response and host defense process [76,77]. However, persistent or inappropriate TNF-α expression causes serious consequences, such as apoptosis and septic shock [78]. Under microgravity conditions, the suppression of TNF-α in microgravity-induced macrophage malfunction has been widely reported. However, the mechanism by which microgravity exposure affects the expression of TNF-α in macrophages remains to be fully clarified.

#### 3.1.1. TNF-α Expression under Simulated Microgravity Conditions

Microgravity exposure inhibits the expression of TNF-α in macrophages. At protein levels, the production of TNF-α is inhibited in macrophages under simulated microgravity conditions. In a simulated microgravity (RCCS) experiment, the expression of TNF-α in LPS-stimulated the murine macrophage cell line (RAW264.7) was lower (100 ng/mL, *p* < 0.001) than that in the 1 g control [16]. However, in the absence of LPS, the expression of TNF-α was almost the same. Furthermore, the same results were also obtained in freshly isolated primary mouse macrophages. These results implied that microgravity exerts a negative impact on the expression of TNF-α in macrophages. At mRNA levels, interestingly, simulating microgravity did not influence the TNF-α mRNA stability. However, due to the lack of sufficient experimental data in microgravity, the TNF-α mRNA expression in macrophages has not been fully elucidated.

So far, there is no evidence that the TLR4/NF-kB signaling pathway is involved in microgravity-induced TNF-α suppression in macrophages. Macrophages stimulated by LPS (lipopolysaccharide) can mimic the inflammation and release inflammatory cytokines such as NO and TNF-α. In the initial stage of signal transduction, there was no significant difference in the expression of TLR4 (Toll-like receptors 4) in LPS-stimulated macrophages under simulated microgravity and normal gravity [16]. In the downstream signaling pathway, the activation of NF-kb protein in macrophages was modest in both normal gravity and simulated microgravity. In general, phosphorylation of NF-kB kinase (IKK) and c-Jun N-terminal kinase (JNK) represents the activation of NF-kb and MAPK pathways, respectively. Subsequently, the dephosphorylated NF-kB and JNK proteins enter the cell nucleus and bind to specific sequences, eventually leading to the release of TNF-α [79]. Under simulated microgravity conditions, the phosphorylation amounts of IKK and JNK in macrophages were not significantly different from that of the 1 g control. Moreover, no obvious difference was observed in LPS-induced nuclear translocation of NF-kB in both simulated microgravity and normal gravity. This was consistent with an earlier report that microgravity exposure did not affect the nuclear translocation of NF-kB [80]. It is worth noting that TLR4 also functions through the endocytosis TRIF (Toll-interleukin 1 receptor-domain-containing adapter-inducing interferon-b) -TRAM (TRIF-related adaptor molecule) pathway [81], which raises a question: Is the endocytosis TRIF-TRAM pathway also affected by microgravity? Unfortunately, to the best of our knowledge, the data on the effects of microgravity on the endocytosis TRIF-TRAM pathway has not been reported yet, which may be an interesting direction for future study. Overall, the above data indicated that simulated microgravity does not affect the TLR4/NF-kb signaling pathway.

At present, several studies have reported that the expression of TNF-α in macrophages is related to temperature [82]. Under normal gravity conditions, the inhibition of febrile range temperature-mediated TNF-α expression is as effective as the soluble inhibitor of TNF-α (glucocorticoids, IL-6) [83]. Moreover, the overexpression of heat shock factors 1 (HSF-1) is involved in the negative regulation of high temperature-caused TNF-α expression. As a transcription repressor of TNF-α promoter, HSF-1 is constitutively expressed in most cells and plays a key role in protecting organisms from severe stress. Under a non-stressed state, HSF-1 is located in the cytoplasm and acts as a transcription factor in heat shock response. Under stress state, HSF-1 enters the nucleus rapidly, activates the heat shock protein transcription, and resists the heat destruction [84].

**Figure 1:**
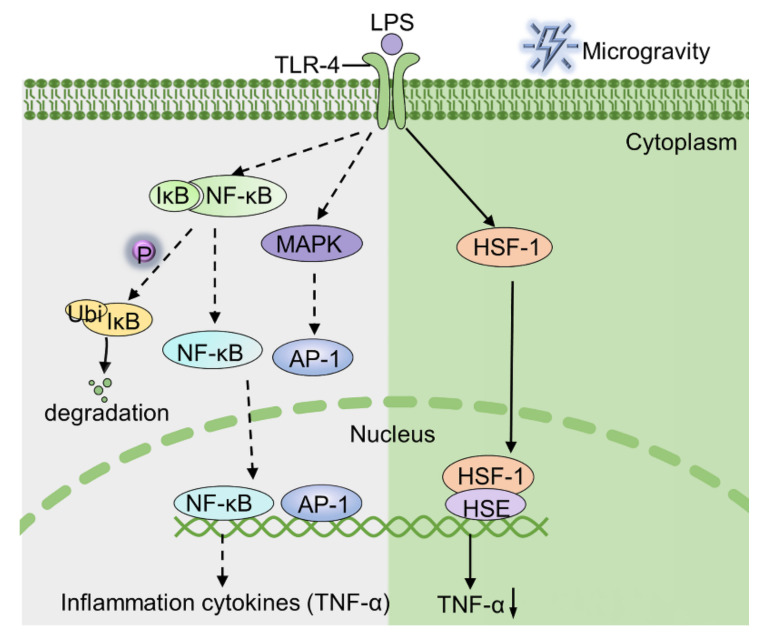
The mechanism by which LPS stimulates macrophages to produce TNF-α under normal gravity and microgravity conditions. The green part (right) illustrates that the binding of HSF-1 to the TNF-α promoter region is responsible for the depression of TNF-α. AP-1, activator protein 1; HSE, heat shock element; IkB, inhibitor of nuclear factor kappa-B.

Under simulated microgravity conditions, the activation of HSF-1 attenuates TNF-α production in LPS-stimulated macrophages [82]. Under 1 g conditions, it was difficult to detect the expression of HSF-1 in nuclear extracts of LPS-stimulated macrophage. In contrast, large amounts of HSF-1 was detected in nuclear extracts of LPS-stimulated macrophages under simulated microgravity conditions [16]. However, the mechanism by which simulated microgravity activated the expression of HSF-1 is yet unknown. In our opinion, activated HSF-1 binds to the heat shock response element-like sequence of the TNF-α promoter region, which in turn inhibits the expression of TNF-α in LPS-stimulated macrophages [85]. Taken together, microgravity exposure induces nuclear translocation of HSF-1, which subsequently suppresses TNF-α secretion in LPS-stimulated macrophages (Figure 1).

#### 3.1.2. TNF-α Expression in Real Microgravity

Unfortunately, there are few studies aimed to investigate the expression of TNF-α in real microgravity. So far, the results from the blood test of astronauts indicate that spaceflight negatively regulates the expression of TNF-α [41,46]. However, the expression of TNF-α in murine B6MP102 cells was upregulated [38]. Overall, the mechanism of TNF-α expression in space still needs further study to be clarified.

### 3.2. Arginase I

Arginase I, a pivotal element in the urea cycle, is highly expressed in M2 macrophages [86,87]. In addition to participating in the urea cycle, arginase I also plays a crucial role in immune regulation [88,89]. As early as the 1980s, studies have revealed that activated macrophage-secreted arginase I orchestrates immune responses by consuming l-arginine in the microenvironment [90]. Currently, arginase I exerts its immune function mainly in two ways. On the one hand, arginase I mediates the consumption of l-arginine, thereby limiting the production of antibacterial NO catalyzed by inducible NO synthase. On the other hand, arginase I inhibits T cell-mediated immune response by down-regulating the TCRζ chain. Therefore, immune regulation mediated by arginase I cplays an important role in the process of anti-inflammatory responses and wound healing.

#### 3.2.1. Arginase I Expression under Simulated Microgravity Conditions

Simulated microgravity up-regulates the expression of arginase I in macrophages. Under simulated microgravity conditions (RCCS), the expression of arginase I was significantly higher compared with 1 g control in primary peritoneal macrophages [17]. The regulatory mechanisms of arginase I expression have been previously reported in detail. So far, two molecules are involved in up-regulation of arginine-1 expression: (1) STAT6 (signal transducer and activator of transcription 6) [91]; (2) C/EBPβ (CCAAT-enhancer-binding proteins β) [92].

- STAT6

STAT6, a member of the STAT family, is involved in intracellular signal transduction and transcriptional activation. During the activation of the STAT6 signaling pathway, extracellular stimuli lead to the phosphorylation of STAT6. Subsequently, phosphorylated STAT6 translocates to the nucleus, binds with the specific sequence, and then initiates gene transcription. However, there was no marked difference in the amounts of phosphorylated STAT6 under the two gravity conditions (microgravity and 1 g). In addition, the expression of YM-1 (regulated by STAT6 activation) in simulated microgravity was also not significantly different from that of the 1 g control group. These results demonstrated that STAT6 is not involved in microgravity-caused arginase I augment [17].

- C/EBPβ

C/EBPβ is one of the crucial transcription factors regulating gene expression of monocytes and is highly expressed in myeloid cells and macrophages [93]. C/EBPβ anticipates in the modulation of multiple aspects of monocytes, including proliferation, differentiation, and immune response [94]. Under simulated microgravity (RCCS) conditions, higher secretion of C/EBPβ in primary mouse peritoneal macrophages was detected in comparison with 1 g control (*p* < 0.05). Furthermore, enhanced arginase I expression was also found at the same time. Overall, microgravity exposure enhances the expression of C/EBPβ in macrophages, which subsequently leads to cup-regulation of arginase I expression.

Microgravity exposure upregulates the expression of C/EBPβ in macrophages via the p38-MAPK pathway [17]. At present, the mechanisms by which microgravity activates the expression of C/EBPβ have been revealed, which involves a series of signaling molecules, including p38-MAPK, JNK, and ERK (extracellular regulated protein kinase). The above three molecules (JNK, p38-MAPK, and ERK) all belong to the MAPK family and are activated by different extracellular stimuli through independent signaling pathways [95]. Interestingly, only p38-MAPK activation was detected in macrophages under simulated microgravity conditions and it was higher than the 1 g control. In addition, there was no significant difference in the expression of JNK and ERK in macrophages in both 1 g and microgravity groups. To further identify the effect of p38 MAPK activation on the expression of C/EBPβ and arginase I in macrophages, anisomycin (activator of p38 MAPK and JNK) and SP600125 (JNK inhibitor) were used to specifically activate the p38 MAPK pathway. These results showed that the specifically activated p38 MAPK did increase the expression of C/EBPβ and arginase I in macrophages, and this effect could be reversed by a p38 inhibitor (SB202190).

**Figure 2:**
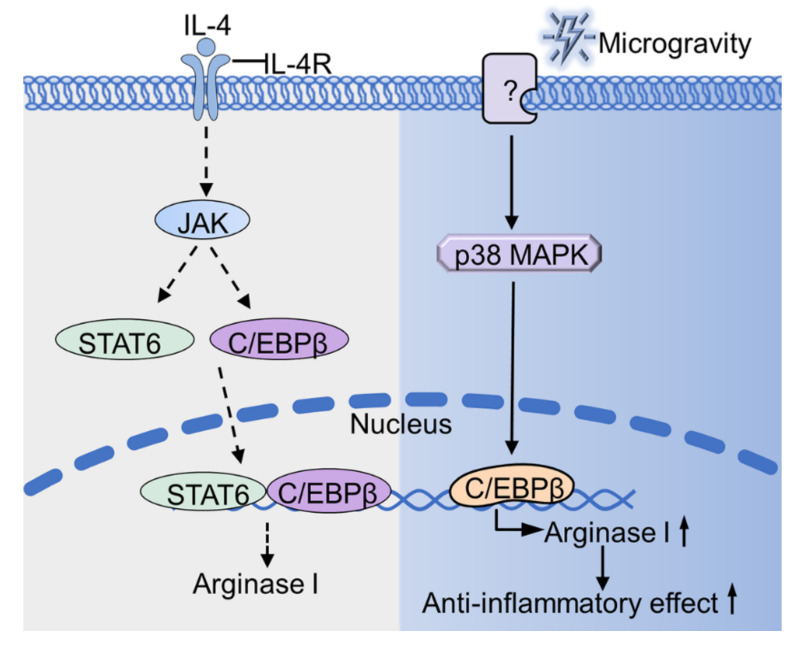
Microgravity regulates the expression of arginase I. Microgravity up-regulates the expression of arginase I through p38 MAPK-C/EBPβ pathway. The unknown protein (indicated as “?”) senses microgravity and transmits the signal to p38 MAPK, thus causing a series of cascade conduction (as shown in the blue part). The gray part (as shown on the left) indicates the signal pathway of IL-4 stimulated cells under normal gravity. IL-4R, interleukin-4 receptor; JAK, Janus kinase; YM1, Chitinase-like protein.

Collectively, myeloid cells-expressed arginase I cexerts a powerful anti-inflammatory effect [96]. Through the p38 MAPK-C/EBPβ signaling pathway, microgravity up-regulates the expression of arginase I (Figure 2), which would be one of the mechanisms of immune dysfunction under microgravity conditions.

#### 3.2.2. Arginase I Expression in Real Microgravity

Unfortunately, to the best of our knowledge, the report on the expression of arginase I in real microgravity has not been found yet.

### 3.3. ICAM-1

**Figure 3:**
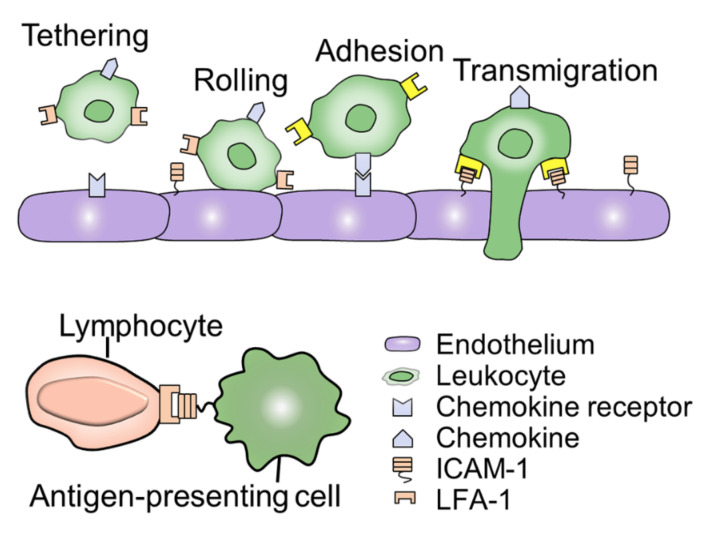
ICAM-1 mediated cell-cell interaction. The process of ICAM-1-mediated leukocyte migration and adhesion are essential for cell communication and connection. In the process of leukocyte migration, LFA-1 is activated after chemokines bind to receptors on endothelial cells, as shown in the yellow part. Besides, ICAM-1 is also involved in the interaction between immune cells and is an important element of the body’s defense.

Adhesion is the basis of leukocytes in immune and inflammatory responses and is necessary for leukocyte-mediated cytotoxicity, phagocytosis, and chemotaxis [75]. The process of adhesion is transient and usually occurs after cell activation, which is mediated by a family of cell surface adhesion molecules [97]. ICAM-1 is a member of the cell surface adhesion molecule family and is widely expressed in macrophages [98]. ICAM-1 plays an important role in mediating the interaction between immune cells, such as antigen presentation (Figure 3). Recently, a series of reports show that microgravity exposure changes the expression of ICAM-1 in macrophages. However, the role of ICAM-1 in the process of macrophages responding to simulated microgravity is not clear yet. Given that the immune function is dysregulated in microgravity, investigating the expression of ICAM-1 will be helpful in exploring the effects of microgravity on cell-cell interactions.

#### 3.3.1. ICAM-1 Expression under Simulated Microgravity Conditions

The response of ICAM-1 to simulated microgravity conditions varies with different experimental systems. At present, a few experiments are focusing on the expression of ICAM-1 in macrophages under simulated microgravity conditions. However, the above experimental results did not reach a unanimous conclusion. Murine BV-2 cells cultured in 2D-clinostat (60 rpm) showed decreased ICAM-1 expression, while U937 macrophages exhibited increased expression of ICAM-1 under the same experimental conditions [99]. Due to the valuable experimental opportunities in this area, more data is needed to reach a consistent conclusion. Overall, the expression of ICAM-1 varies with different cell types under simulated microgravity conditions.

#### 3.3.2. ICAM-1 Expression in Real Microgravity

The responses of ICAM-1 to real microgravity is a cell-specific process. At the protein level, the expression of ICAM-1 under microgravity conditions (sounding rocket) was marked lower (*p* < 0.005) than that of the 1 g control in human primary M2 macrophages (PromoCell) [91]. Conversely, the expression of ICAM-1 was up-regulated in differentiated U937 cells in real microgravity (Shenzhou-8, 5d). At the mRNA level, interestingly, the mRNA expression of ICAM-1 remained essentially unchanged in these cells. These findings are consistent with the mRNA expression of ICAM-1 in endothelial cells under microgravity conditions (or returned to normal level after 24 h) [100]. At the signaling pathway level, microgravity exposure for 20 s caused a significant change in natural killer cell-mediated cytotoxicity. However, this effect was reversed after 6 min of microgravity exposure. Collectively, the above evidence suggested that the response of natural killer cells to microgravity is a short-term, recoverable process. Interestingly, the authors of the above studies proposed that the NF-kB signaling pathway remained unchanged under microgravity conditions, which is consistent with the earlier result [16].

Inflammatory factors promote the expression of ICAM-1 in macrophages. Compared with 1 g control, the expression of ICAM-1 was significantly up-regulated in PMA-stimulated BV-2 cells in spaceflight. In addition, TNF-α slightly blocked the microgravity-induced ICAM-1 inhibition [99]. It is worth noting that the different dose of TNF-α causes diametrically opposite experimental results. For example, normal concentrations of TNF-α block microgravity-induced ICAM-1 suppression, while high concentrations of TNF-α cause apoptosis. In the same line, another experiment regarding macrophages under 1 g conditions showed the expression of ICAM-1 (*p* < 0.05) was inhibited by Dexamethasone, while it was enhanced by IFN-γ, IL-1, and TNF-α (*p* < 0.01) [101]. Altogether, these findings would be a useful clue for drug intervention to regulate the expression of ICAM-1 in space.

As for the discrepancy of ICAM-1 expression in different experimental systems under microgravity conditions, there is no consistent conclusion yet. We speculate that this phenomenon may be caused by the following factors: (1) Limited experimental opportunities; (2) different biological experiment systems, such as sample types and treatment methods; (3) poor consistency in the space environment, such as space radiation, circadian rhythms, and vibration.

The cytoskeleton is a complex network of fibers that are sensitive to gravity. It is noteworthy that the regulation of ICAM-1 expression is a highly dynamic process, which is related to the function of the cytoskeleton. Interestingly, the composition of the cytoskeleton is markedly changed in both real microgravity and simulated microgravity [15,102,103,104]. However, the mechanism by which microgravity exposure affects the cytoskeleton has not yet been completely understood. In conclusion, ICAM-1-mediated intercellular communication and connection are essential for the immune system to respond to external signals. Under microgravity conditions, the expression of ICAM in macrophages is significantly changed, which is one of the causes of macrophage dysfunction.

## 4. Discussion

### 4.1. Molecules in Macrophages Sensitive to Real and Simulated Microgravity

The impact of weightlessness on immune function, which previously received little attention, is becoming an emerging field. It has been over 40 years since the dysfunctional immune system in weightlessness was first reported. With the extensive study of the immune system in space, increasing evidence has shown that microgravity exposure causes severe abnormalities in immune cells. Specifically, the activity of T cells to mitogens, the cytotoxic activity of NK cells, and the response of bone marrow cells to macrophage colony-stimulating factors are attenuated during spaceflight [105]. It is noteworthy that several studies note that the decreased production of IL-1 should be accounted for by the impaired activation of lymphocytes [106]. However, the underlying cellular and molecular mechanisms of microgravity-induced immune dysfunction have not been fully addressed.

Macrophage polarization plays an essential role in macrophage dysfunction under microgravity conditions. However, the effect of microgravity on macrophage polarization has not been elucidated. In this review, three molecules in macrophages that respond to the microgravity environments are systematically summarized: TNF-α, arginase I, and ICAM-1.

At the level of protein expression, microgravity down-regulates the expression of TNF-α and up-regulates the expression of arginase I in macrophages [16,17]. As for ICAM-1, microgravity exposure affects the expression of ICAM-1 in macrophages. However, the expression of ICAM-1 has not been unified, as some cells (U937 macrophages) exhibit an increase in ICAM-1 expression, while other cells (BV-2 cells) show a decreased expression [99]. At the level of signaling pathway, p38 MAPK-C/EBPβ pathway is involved in up-regulation of arginase I [17]. Besides, microgravity decreases the expression of TNF-α by increasing the expression of HSF-1 [16]. At the functional level, TNF-α is known to be highly expressed in M1 macrophages, while arginase I is highly expressed in M2 macrophages [107]. Moreover, M1 macrophages exert pro-inflammatory and pathogen clearance functions, and M2 macrophages have anti-inflammatory and tissue repairing effects [108]. Therefore, decreased expression of TNF-α or increased expression of arginase I lead to immunosuppression in weightlessness, which subsequently affects the phagocytosis effect and oxidative burst reaction of macrophages.

**Figure 4:**
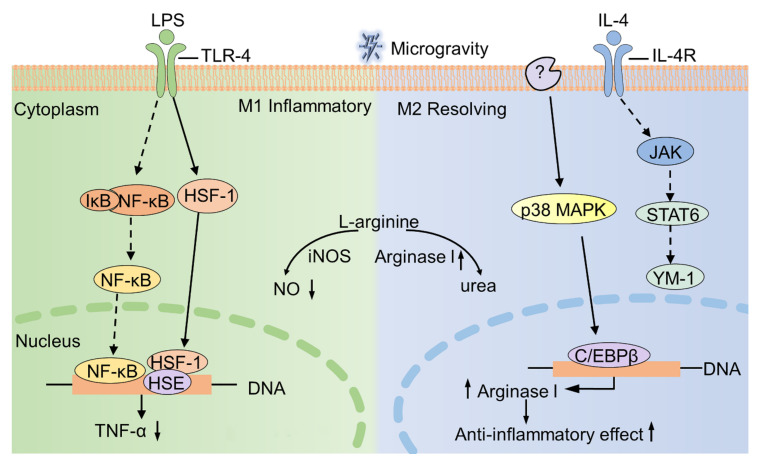
The effect of microgravity on macrophage polarization. M1 macrophages (**left**) are induced by LPS and highly express pro-inflammatory factors (TNF-α). M2 macrophages (**right**) are activated by IL-4 and are characterized by high expression of arginase I. Under microgravity conditions, HSF-1 and C/EBPβ are activated, resulting in a decrease of TNF-α expression and an increase of arginase I expression, respectively. Solid lines indicate the pathway affected by microgravity, whereas dashed lines indicate the pathway is not affected by microgravity. iNOS, inducible nitric oxide synthase.

Moreover, the l-arginine metabolism pathway is also affected by microgravity (Figure 4). Due to the increased expression of arginase I, the production of NO via INOS (l-arginine as a substrate) may be decreased to some extent. Interestingly, microgravity exposure indeed causes the suppression of NO production in macrophages, although the mechanism remains unclear [12]. Taken together, these alterations in macrophage function are the reasons that result in immune system disorders in spaceflight.

### 4.2. The Impact of Real and Simulated Microgravity on Immune Cells

The impact of microgravity on all immune cells is a topic worthy for further exploration. Under microgravity conditions, the TNF-α levels of macrophages were significantly decreased compared with the control group. It may be possible that decreased TNF-α is a consequence of having fewer monocytes/macrophages in the blood, which in turn could be caused by a defect in bone marrow physiology. As the mother of almost all immune cells, the response of bone marrow to microgravity stimulation may have lasting effects on any cells that are derived from it, including monocyte production, epigenetic imprinting, and monocytosis. Studies by Kaur et al. showed that the number of monocytes in the crew member became higher after the 5-day mission compared with the 10-day preflight. Moreover, three days after landing, the number of monocytes in the crew member returned to the level before the flight [61]. However, there is no consistent conclusion on the influence of microgravity on the number of monocytes in current studies, as shown in Table 2. Overall, the impact of microgravity on bone marrow is a profound and complicated process, which is worthy of further study.

### 4.3. The Problems of Simulation of Microgravity in Comparison to Real Microgravity

The limitation of the ground-based microgravity device is also a concern. Although a variety of microgravity simulation facilities are widely used in biological and medical research, such devices still lead to inconsistent experimental results. One of the most important and critical points is that ground-based models can not fully restore space conditions, such as radiation, stress, vibration, circadian rhythm, and many other undetermined factors. These factors may also be involved in spaceflight-caused immune dysfunction [7]. Moreover, most of the current studies are conducted at the level of a single cell type, while few studies focus on the interaction between different types of cells and their integration in vivo. For example, microgravity-induced changes in calcium utilization in musculoskeletal systems have a significant impact on the immune system [109]. Therefore, the above discrepancies may contribute to the inconsistent results (Table 1 and Table 2).

In studying the response of various biological tissues to microgravity by simulating microgravity conditions, different artificial microgravity simulation devices have several notable shortcomings. Firstly, the volume of various devices that simulate microgravity is limited, and there is a certain gap between the quality of such simulated microgravity and real microgravity. Secondly, when the experimental results obtained under different simulated microgravity conditions are compared, the lack of specific technical details (such as the direction, speed, and speed of rotation) makes it difficult to evaluate the consistency of such experimental results. Finally, the details of the hardware (dimensions, materials, and location within the device) are often missing, which often causes inconsistencies and even errors in the interpretation of experimental results [22].

Therefore, when ground-based microgravity experiments are carried out, the following items need to be carefully considered: (1) The biological state of the sample (such as gender, strain, and age) should be carefully considered in the experimental design stage; (2) ground-based experiments should be carried out to maximize the consistency of the experimental results between simulated microgravity and real microgravity so that the final consistent conclusion can be obtained; (3) given the sensitivity of biological samples to the external environment, the container of the device that simulates microgravity should be the same size as the container in the real space flight experiment system, although this experimental requirement may increase the cost of the real space flight experiment [110,111].

Despite the above deficiencies, research in this field still presents a rapid development trend and is highly promising. On the one hand, with the continuous development of aerospace technology, space experiments have also gained new chances, including increased opportunities, reduced costs, and improved conditions. On the other hand, ground-based simulation technology is continuously improving, which is approaching the real microgravity environment.

### 4.4. Perspective

There is no doubt that the microgravity simulator has become a powerful tool for microgravity biology research. Excitingly, several new microgravity simulation equipment or modules have been developed [112,113], which are positive supplements to existing microgravity simulation equipment. Moreover, a recently reported platform has achieved similar experimental results with existing microgravity simulation equipment [114], which will help greatly promote the progress in the field of simulated microgravity research.

As an emerging player in microgravity, macrophages play a vital role in microgravity-induced immune system dysfunction. In particular, the advancement of space omics research may add new fuel to the research of space life sciences [115]. Although current studies suggest that microgravity affects the function of macrophages, more in-depth exploration is still needed to identify its regulatory mechanisms. In conclusion, investigating the immune system malfunction in space with macrophages provides a meaningful clue for the future study of space biomedicine.

## Abbreviations

2-D clinostat	Two-dimensional clinostat
AP-1	Activator protein 1
BMDM	Bone marrow-derived macrophage
C/EBPβ	CCAAT-enhancer-binding proteins β
Con A	Concanavalin A
HSE	Heat shock element
HSF-1	Heat shock factors 1
ICAM-1	Intercellular adhesion molecule 1
IL-4R	Interleukin-4 receptor
IL-4	Interleukin-4
IL-6	Interleukin-6
IL-8	Interleukin-8
iNOS	Inducible nitric oxide synthase
IkB	Inhibitor of nuclear factor kappa-B
JAK	Janus kinase
LFA -1	Lymphocyte function-associated antigen 1
LPS	Lipopolysaccharide
MAPK	Mitogen-activated protein kinase
NF-kB	Nuclear factor-kappa B
NO	Nitric oxide
PBMC	Peripheral blood mononuclear cells
PHA	Phytohemagglutinin
RCCS	Rotary cell culture system
RPM	Random positioning machine;
RWV	Rotating wall vessel
STAT	Signal transducer and activator of transcription
TLR4	Toll-like receptors 4
TNF-α	Tumor necrosis factor α
YM1	Chitinase-like protein
